# Two sides of the same coin? Prevalence and co-occurrence of binge eating disorder and compulsive sexual behavior disorder in a representative sample of the Polish population

**DOI:** 10.1093/sexmed/qfaf092

**Published:** 2025-11-09

**Authors:** Ewelina Kowalewska, Michał Lew-Starowicz

**Affiliations:** Department of Psychiatry, Centre of Postgraduate Medical Education, 01-813 Warsaw, Poland; Department of Psychiatry, Centre of Postgraduate Medical Education, 01-813 Warsaw, Poland

**Keywords:** binge eating disorder, compulsive sexual behavior, comorbidity, mental disorders

## Abstract

**Background:**

Although increasingly recognized, research into the prevalence and co-occurrence of binge eating disorder (BED) and compulsive sexual behavior disorder (CSBD) remains scarce.

**Aim:**

To evaluate the prevalence and co-occurrence of BED and CSBD in a representative Polish population while examining their associations with sexual behavior–related variables, and levels of anxiety and depression.

**Methods:**

Data were collected from a representative sample of Polish adults, consisting of 1527 participants aged 18-65.

**Outcomes:**

The occurrence and intersection of symptoms of BED and CSBD.

**Results:**

Men reported significantly higher CSBD symptoms (*Z* = 9.62, *P* < .001, *d* = 0.52) and problematic sexual behaviors, while women reported higher BED (*Z* = 5.51, *P* < .001, *d* = 0.30) and anxiety symptoms (*Z* = 7.46, *P* < .001, *d* = 0.39). BED and CSBD symptoms co-occurred significantly in men (*χ*^2^(1) = 59.00, *P* < .001), but not in women (*χ*^2^(1) = 1.51, *P* = .22). BED symptoms were positively correlated with anxiety (*r* = 0.46, *P* < .001) and depression (*r* = 0.47, *P* < .001), highlighting their interplay with psychological distress.

**Clinical Implications:**

Findings underscore shared mechanisms across BED and CSBD, suggesting the potential effectiveness of transdiagnostic treatment approaches addressing co-occurring symptoms, emotional regulation, and impulsivity within a unified therapeutic framework.

**Strengths and Limitations:**

Strengths include a representative sample and the examination of underexplored comorbidity patterns. Limitations include the cross-sectional design and reliance on self-report measures, warranting longitudinal and multimethod research for causal inferences.

**Conclusion:**

This study reveals significant gender differences in BED and CSBD symptoms and demonstrates their co-occurrence among men, emphasizing the need for gender-sensitive, integrated clinical approaches to assessment and treatment.

## Introduction

Eating and sexual behavior are evolutionarily driven forces essential for species survival. At the neurophysiological level, these behaviors represent “primary rewards” within the brain’s reward system, progressing through phases of wanting, liking, and satiety, regulated by dopamine, endogenous opioids, and serotonin.^1^^,^^2^ However, in some individuals, these behaviors can become dysregulated and “out of control,” leading to patterns of binge eating or compulsive sexual behavior.

Binge eating disorder (BED) and compulsive sexual behavior disorder (CSBD) are recognized as significant public health concerns and have been included in the 11th revision of the International Statistical Classification of Diseases and Related Health Problems (ICD-11).^3^ According to ICD-11 criteria (6B82), BED involves recurrent episodes of binge eating—typically at least once per week over several months—characterized by consuming unusually large quantities of food with a perceived loss of control, often accompanied by feelings of guilt, shame, or disgust. Unlike bulimia nervosa, these episodes are not followed by compensatory behaviors such as vomiting or excessive exercise, yet they cause marked distress and impair functioning in various life domains.^3^ BED is the most common eating disorder globally, with lifetime prevalence rates of 2.8% in the United States and 1.9% worldwide, affecting women more frequently than men.^4^^,^^5^ Despite its prevalence and the associated risk of conditions such as obesity, diabetes, and hypertension,^6^ BED often goes undiagnosed in healthcare settings. Comorbid psychiatric conditions, including mood and anxiety disorders, substance use disorders, and impulse control difficulties, are frequently observed, complicating treatment and underscoring the disorder’s wide-ranging impact on mental health.^7^

CSBD (ICD-11: 6C72) is characterized by a persistent inability to control intense, repetitive sexual urges and behaviors over a period of at least 6 months, leading to significant distress or impairment in personal, social, or occupational functioning. Importantly, distress arising solely from moral or societal disapproval does not qualify for this diagnosis.^3^ Individuals with CSBD may continue engaging in sexual behaviors despite adverse consequences and attempts to reduce or control their actions. Estimates suggest that 4.9% of men and 3% of women meet the ICD-11 criteria for CSBD.^8^^,^^9^ Similar to BED, CSBD is often comorbid with psychiatric conditions such as depression, anxiety, and substance use disorders, further complicating clinical presentation and treatment.^10^^,^^11^

Although classified as distinct disorders, BED and CSBD may reflect overlapping dimensional traits within a broader psychopathological continuum. Dimensional models of psychopathology propose that symptoms exist along spectra of severity, with both disorders sharing underlying vulnerabilities such as reward dysregulation and impulse control deficits. Neurobiologically, these shared traits are linked to alterations in the mesolimbic dopamine pathway, where heightened sensitivity to rewarding stimuli (eg, food or sexual content) can drive compulsive behaviors despite negative consequences. Structural and functional changes in brain regions governing reward processing, including the ventral striatum and orbitofrontal cortex, have been documented in both BED and CSBD.^12-15^

Despite growing recognition of these conditions and their potential overlap, research directly examining the prevalence and co-occurrence of BED and CSBD remains limited. The present study aims to address this gap by assessing the prevalence of BED and CSBD and exploring their co-occurrence within a representative sample of the Polish population. Additionally, we examine associations between BED and variables related to sexual behavior, as well as symptoms of anxiety and depression, which are commonly comorbid with both BED and CSBD.^7^^,^^10^^,^^11^ The selection of these disorders for comparison is motivated by their shared clinical phenomenology and underlying mechanisms, particularly the loss of control over evolutionarily significant and rewarding behaviors. By shedding light on the prevalence and interrelationship between these conditions, this research aims to inform clinical practice and public health policy, supporting the development of integrated, gender-sensitive, and transdiagnostic treatment approaches.

*Hypotheses.*

(H1) Given their similar risk factors and psychopathological underpinning, we expect that CSBD and BED symptoms often co-occur (ie, “two sides of the same coin”).

(H2) We assume there will be a positive correlation between both CSBD and BED and the severity of anxiety and depressive symptoms.

(H3) Gender differences are expected regarding CSBD and BED prevalence and comorbidities.

## Materials and methods

### Procedure and participants

To ensure accuracy and generalizability, data from a representative sample of the Polish adult population were collected via Pollster (https://pollster.pl/), a Polish market-research institute. We employed a CAWI (computer-assisted web interviewing) approach using Pollster’s online panel, whose members opt in to periodic surveys and whose sociodemographic profiles are verified to mirror the adult Polish population (age, gender, education, relationship and employment status, and place of residence). Quota sampling ensured that the sample distribution matched national census benchmarks across key strata. Invitations were sent to panel members meeting prespecified criteria, with reminders and attention checks integrated to maximize data quality. Respondents provided informed consent electronically before beginning the questionnaire, and all the procedures adhered to relevant ethical guidelines and data-protection regulations.

The final sample consisted of 51.5% women (*n* = 787) and 48.5% men (*n* = 740) aged between 18 and 65 years (*M*_age_ = 41.63; SD_age_ = 13.09). All the sample sociodemographic and health-related characteristics are presented in Table 1.

**Table 1:** Demographic and health-related factors divided by gender.

	**Women** **(n = 787)**	**Men** **(n = 740)**
	***M* (SD)/n (%)**	
**Age**	40.06 (SD = 13.26)	43.29 (SD = 12.72)
**Age in intervals** 18-24 25-34 35-44 45-54 55 and more	105 (13.3) 189 (24) 206 (26.2) 136 (17.3) 151 (19.2)	66 (8.9) 133 (18) 182 (24.6) 183 (24.7) 176 (23.8)
**Relationship status** Married Informal relationship During divorce or separation Single	355 (45.1) 225 (28.6) 49 (6.2) 158 (20.1)	347 (46.9) 141 (19.1) 38 (5.1) 214 (28.9)
**Occupation** Full-time Student, unemployed Student, employed Unemployed	454 (57.7) 51 (6.5) 46 (5.8) 236 (30)	542 (73.2) 26 (3.5) 22 (3) 150 (20.3)
**Education level** Primary school Vocational school High school College (still at school) Graduate degree Postgraduate degree	56 (7.1) 215 (27.3) 242 (30.7) 26 (3.3) 247 (31.4) 1 (0.1)	41 (5.5) 207 (28) 264 (35.7) 14 (1.9) 210 (28.4) 4 (0.5)
**Place of residence** Village City up to 10 000 City from 10 000 to 50 000 City from 50 000 to 100 000 City from 100 000 to 500 000 City over 500 000	318 (40.4) 37 (4.7) 117 (14.9) 87 (11.1) 108 (13.7) 120 (15.2)	272 (36.8) 43 (5.8) 126 (17) 105 (14.2) 124 (16.8) 70 (9.5)
**Using psychological support in the past** Psychologist occasionally (>1 per month) Individual psychotherapy Group psychotherapy Support group Other None of the above	116 (14.7) 106 (13.5) 25 (3.2) 17 (2.2) 12 (1.5) 557 (70.8)	81 (10.9) 75 (10.1) 50 (6.8) 33 (4.5) 7 (0.9) 558 (75.4)
**Using psychological support currently** Psychologist occasionally (>1 per month) Individual psychotherapy Group psychotherapy Support group Other None of the above	26 (3.3) 31 (3.9) 3 (0.4) 6 (0.8) 3 (0.4) 723 (91.9)	39 (5.3) 27 (3.6) 8 (1.1) 12 (1.6) 2 (0.3) 664 (89.7)
**Height**	165.03 (SD = 9.98)	177.49 (SD = 12.43)
**Weight**	69.48 (SD = 17.37)	85.03 (SD = 18.51)
**Are you undergoing treatment for obesity?** Yes, I am taking medication Yes, I have undergone surgery No, but I am planning to undergo treatment I have not undergone treatment and do not plan to Not applicable	9 (1.1) 5 (0.6) 39 (5) 397 (50.4) 337 (42.8)	6 (0.8) 11 (1.5) 28 (3.8) 379 (51.2) 316 (42.7)
**Smoking cigarettes** Regular E-cigarette^a^ Nonsmoker	216 (27.4) 63 (8) 525 (66.7)	272 (36.8) 71 (9.6) 425 (57.4)
**Daily number of cigarettes smoked** Regular E-cigarette	12.85 (SD = 8.12) 9.54 (SD = 14.18)	26.08 (SD = 183.61) 19.27 (SD = 62.31)
**Sexual orientation** Exclusively heterosexual Predominantly heterosexual, only incidentally homosexual Predominantly heterosexual, but more than incidentally homosexual Equally heterosexual and homosexual Predominantly homosexual, but more than incidentally heterosexual Predominantly homosexual, only incidentally heterosexual Exclusively homosexual	658 (83.6) 47 (6) 12 (1.5) 9 (1.1) 4 (0.5) 7 (0.9) 50 (6.4)	652 (88.1) 30 (4.1) 8 (1.1) 7 (0.9) 6 (0.8) 6 (0.8) 31 (4.2)

^a^In the case of e-cigarettes, the number of cigarettes was estimated based on the assumption that “one cigarette” corresponds to approximately 15 puffs or lasts about 5 minutes.

### Measures

Participants provided demographic and health-related information, including age, relationship status, occupation, education level, size of the place of residence, and sexual orientation. Additional data were collected on participants’ height, weight, history of obesity treatment, use of support services (past and current), and smoking status (see Table 1).

The Polish version of the Binge Eating Scale (BES)^16^ was used to assess the severity of BED symptoms. This 16-item questionnaire captures behavioral aspects such as rapid eating and excessive food consumption, as well as cognitive and affective dimensions like guilt and perceived loss of control. Each item offers three to four response options reflecting increasing severity. For example: “1. I don’t feel self-conscious about my weight or body size when I’m with others; 2. I feel concerned about how I look to others, but it normally does not make me feel disappointed with myself; 3. I do get self-conscious about my appearance and weight which makes me feel disappointed in myself; 4. I feel very self-conscious about my weight and frequently feel intense shame and disgust for myself. I try to avoid social contacts because of my self-consciousness.” The total BES score ranges from 0 to 46, with higher scores indicating more severe binge eating. Established cut scores^17^ classify individuals into three severity groups: no or minimal binge eating (≤17), mild to moderate binge eating (18-26), and severe binge eating (≥27). The Polish version was created through a forward–backward translation by two independent bilingual researchers, followed by a consensus review. Although this version has not yet been fully validated, the original cutoffs were applied with the understanding that optimal thresholds may vary culturally and require further validation. The BES showed high internal consistency in this study (Cronbach’s *α* = 0.91).

The Polish version of the Compulsive Sexual Behavior Disorder – 19 (CSBD-19)^18^ was used to assess CSBD symptom severity. The scale consists of 19 items that measure the frequency and intensity of sexual urges, behaviors, and associated distress or impairment. Example items include, “Even though my sexual behavior was irresponsible or reckless, I found it difficult to stop.” and “I would rather have had sex than to have done anything else.” Participants rate each item on a four-point Likert scale (1 = strongly disagree, 4 = strongly agree). A total score ranges from 19 to 76, with higher scores indicating a greater presence of CSBD symptoms. A score of 50 points was employed as the cutoff for the CSBD-19 scale, as this is the recommended optimal threshold for identifying individuals at high risk for CSBD.^18^ In the present study, the CSBD-19 demonstrated excellent reliability, with a Cronbach’s alpha coefficient of 0.94.

A polish adaptation of the Brief Pornography Screen (BPS),^19^ a concise 5-item psychometric tool, was used to identify features of problematic pornography use (PPU; a key feature of CSBD), such as impaired control and craving. Participants indicate the frequency of certain behaviors over the past 6 months on a 3-point Likert scale (0 = never; 1 = sometimes; 3 = very often). With scores ranging from 0 to 10, a cutoff score of 4 suggests potential PPU. The scale includes items such as “You find it difficult to resist strong urges to use pornography.” and “You find yourself using pornography to cope with strong emotions (e.g., sadness, anger, loneliness, etc.).” The BPS demonstrated a high level of internal consistency in this study, with a Cronbach’s alpha of 0.88.

A Polish version of the Hospital Anxiety and Depression Scale (HADS) was used to assess anxiety and depression symptoms,^20^ which are frequently comorbid with BES and CSBD and may influence their severity and course. The questionnaire consists of 14 items, divided into 2 subscales: 7 items measuring anxiety (HADS-A) and 7 items measuring depression (HADS-D). An illustrative item from the anxiety subscale is “Worrying thoughts go through my mind,” whereas an example item from the depression subscale is “I still enjoy the things I used to enjoy.” Each item is rated on a 4-point Likert scale, with total scores ranging from 0 to 21 for each subscale. For both the anxiety and depression subscales, scores of 0-7 indicate normal levels, 8-10 reflect a borderline level, while scores of 11 or above are considered pathological. In this study, the HADS exhibited strong internal reliability, as indicated by a Cronbach’s alpha of 0.90.

Additionally, we gathered comprehensive data on various aspects of sexual behavior and related issues. Participants were asked about their difficulty in controlling sexual behavior, including specific types of behaviors they have struggled with and whether they have sought help or are currently using medication for sexual difficulties. Information was collected on the onset of first pornography exposure, types of sexual experiences with partners before pornography, and frequency and duration of pornography use over the past year, including the amount of time spent on it in the last 7 days and the average duration of sessions. The study also explored details about masturbation, such as the age of first masturbation, maximum frequency per day, and current frequency over the past month. Lastly, participants provided information on their first sexual intercourse experience, how they rate that experience, and the number of sexual partners they have had since sexual initiation, in the last year, and in the past 30 days. Information on sexual behavior and related issues was collected using nonstandardized questions based on items commonly used in previous research and clinical practice.

The study procedures were carried out in accordance with the Declaration of Helsinki. The Bioethics Committee of the Centre of Postgraduate Medical Education approved the study. All participants were informed about the scope of the study, and all provided informed and voluntary consent.

### Statistical analysis

All the statistical analyses were conducted using IBM SPSS (version 25.0). Descriptive analyses were initially performed to summarize participants’ sociodemographic characteristics. To compare gender differences in sexual behavior variables and questionnaire scores, we applied the Mann–Whitney *U* test for continuous variables and Pearson’s chi-square test for categorical variables. The same analyses were used to compare sexual behavior and related factors between participants scoring below and above the suggested cutoff value on the BES questionnaire. Effect sizes, including Cohen’s *d* and Cramer’s *V*, were calculated using G^*^Power (version 3.1). Additionally, Pearson correlations were conducted to examine associations among scores from all questionnaires. Finally, the prevalence and co-occurrence of BED and CSBD symptoms were assessed using Pearson’s chi-square test.

## Results

A gender-based comparison of variables related to sexual behavior is outlined in Table 2. A significantly higher proportion of men, compared to women, reported difficulties in controlling their sexual behavior (*χ^2^*(1) = 26.25, *P* < .001, *ϕ_c_* = 0.13). Among out-of-control sexual behaviors, men were notably more likely to report pornography consumption (*χ^2^*(1) *=* 20.26, *P* < .001, *ϕ_c_* = 0.29), excessive masturbation (*χ^2^*(1) = 8.34, *P* < 0.01, *ϕ_c_* = 0.18), and using erotic chats or sex cameras (*χ^2^*(1) = 8.48, *P* < .01, *ϕ_c_* = 0.19).

**Table 2:** Various aspects of sexual behavior and related factors divided by gender.

	**Women** **(n = 787)**	**Men** **(n = 740)**	**Mann–Whitney *U* (Cohen’s d)**	***χ*** ^***2***^ **(Cramér’s *V*)**
**Have you ever had difficulty controlling your sexual behavior? (n, %)** Yes No	90, 11.4% 697, 88.6%	156, 21.1% 584, 78.9%	–	26.25^***^ (0.13)
**Types of out-of-control behavior (yes/no) (n, %)** Pornography consumption Excessive masturbation Use of paid sexual services Casual sexual activity Excessive sexual thoughts Erotic chats and cameras Other	**n = 90** 28, 31.1% 33, 36.7% – 16, 17.8% 51, 56.7% 3, 3.3% 11, 12.2%	**n = 156** 95, 60.9% 87, 55.8% 14, 9% 24, 15.4% 72, 46.2% 24, 15.4% 9, 5.8%	–	20.26^***^ (0.29) 8.34^**^ (0.18) – 0.24 (0.03) 2.52 (0.10) 8.48^**^ (0.19) 3.18 (0.11)
**Have you ever sought any form of help due to a loss of control over sexual behavior? (n, %)** Yes No	3 (0.4) 784 (99.6)	26 (3.5) 714 (96.5)	–	20.09^***^ (0.12)
**Type of help used due to sexual behavior (n, %)** Psychological Sexological Self-help groups Religious Psychiatric Other	**n = 3** 2, 66.7% – – – – 1, 33.3	**n = 26** 9, 34.6% 14, 53.8% 5, 19.2% 7, 26.9% 4, 15.4% 1, 3.8%	–	1.17 (0.20) – – – – 3.64 (0.35)
**Onset of first pornography exposure (M; SD)** Has never watched pornography	**n = 599** 17.67; 6.85 **n *=* 188**	**n = 661** 16.76 7.68 **n *= 79***	5.65^***^ (0.13)	**–**
**Frequency of pornography use in the last year (n, %)** Not at all Once a month or less 2-4 times a month 2-3 times a week 4 times a week or more	**n = 599** 364, 60.8% 152, 25.4% 52, 8.7% 17, 2.8% 14, 2.3%	**n = 661** 167, 25.3% 168, 25.4% 129, 19.5% 111, 16.8% 86, 13%	–	225.01^***^ (0.42)
**Time spent on pornography during the last 7 days (in minutes) (M; SD)** Has not watched pornography last week	**n = 232** 18.24; 50.26 **n *=* 367**	**n = 407** 48.42; 67.70 **n *=* 254**	11.43^***^ (0.51)	–
**On average, how many times have you viewed pornography in the last month? (M; SD)**	**n = 599** 1.16, 3.32	**n = 661** 6.38; 9.98	15.77^***^ (0.70)	–
**Average duration of a single pornography session in the last month (in minutes) (M; SD)**	**n = 206** 16.95; 19.19	**n = 480** 18.48; 18.98	1.91 (0.08)	–
**Sexual experiences with partner before pornography (n, %)** Cuddling/Foreplay Masturbation Vaginal contact (intravaginal) Anal contact (intrarectal) Oral–genital contact (French love) Has had no experience	**n = 599** 147, 24.5% 72, 12% 82, 10.4% 10, 1.7% 40, 6.7% 373, 62.3%	**n = 661** 150, 22.7% 179, 27.1% 80, 12.1% 15, 2.3% 65, 9.8% 360, 54.5%	–	0.60 (0.02) 44.68^***^ (0.19) 0.71 (0.02) 0.58 (0.02) 4.10^*^ (0.06) 7.87^**^ (0.08)
**Age of first masturbation (M; SD)** Has never masturbated	**n= 437** 16.80; 6.11 **n *=* 350**	**n = 605** 14.75; 3.99 **n *=* 135**	7.50^***^ (0.40)	–
**Maximum number of masturbations per day (M; SD)**	**n = 437** 2.22; 1.94	**n = 605** 3.24; 2.66	8.91^***^ (0.44)	–
**Frequency of masturbation in the last month (n, %)** Not at all Once a month or less 2-4 times a month 2-3 times a week 4 times a week or more	**n = 437** 163, 37.3% 134, 30.7% 103, 23.6% 24, 5.5% 13, 3%	**n = 605** 173, 28.6% 96, 15.9% 145, 24% 116, 19.2% 75, 12.4%	–	93.16^***^ (0.30)
**Onset of first sexual intercourse (M; SD)** Never had sexual intercourse	**n = 695** 19.22; 3.76 **n *=* 92**	**n = 636** 19.56; 3.90 **n *=* 104**	1.06 (0.09)	–
**Subjective rate of the first sexual intercourse (from 1 – unsuccessful to 10 – very successful) (M; SD)**	**n = 695** 5.21; 2.87	**n = 636** 6.18; 2.55	6.30^***^ (0.36)	–
**Number of sexual partners since sexual initiation (M; SD)**	**n = 695** 4.78; 7.20	**n = 636** 8.85; 14.94	8.05^***^ (0.35)	–
**Number of sexual partners during the last year (M; SD)**	**n = 695** 0.74; 1.64	**n = 636** 1.16; 3.04	5.10^***^ (0.17)	–
**Number of dyadic sexual intercourse in the last 30 days (M; SD)**	**n = 695** 2.19; 4.45	**n = 636** 2.96; 5.46	3.66^***^ (0.15)	–
**Number of sexual partners in the last 30 days (M; SD)**	**n = 695** 0.73; 1.43	**n = 636** 0.88; 1.67	2.54^**^ (0.10)	–

^***^ *P* < .001; ^**^P < .01; ^*^P < .05

Additionally, a greater number of men than women sought help due to a perceived loss of control over their sexual behavior in the past (*χ^2^*(1) = 20.09, *P* < .001, *ϕ_c_* = 0.12). However, there was no significant gender differences regarding the specific behaviors that prompted help-seeking.

Men also reported an earlier age of exposure to pornography (*Z* = 5.65, *P* < .001, Cohen’s *d* = 0.13), and a higher frequency of pornography use within the past year (*χ^2^*(4) = 225.01, *P* < .001, *ϕ_c_* = 0.42). Men spent more time consuming pornography in the last 7 days (*Z* = 11.43, *P* < .001, Cohen’s *d* = 0.51), and reported more frequent pornography viewing over the last month (*Z* = 15.77, *P* < .001, Cohen’s *d* = 0.70). Men (as compared to women) reported more sexual experiences before their first exposure to pornography, including masturbation (*χ^2^*(1) = 44.68, *P* < .001, *ϕ_c_* = 0.19) and oral–genital contact (*χ^2^*(1) = 4.10, *P* < .05, *ϕ_c_* = 0.06). In contrast, a higher proportion of women than men reported no sexual experience prior to their first exposure to pornography (*χ^2^*(1) = 7.87, *P* < .01, *ϕ_c_* = 0.08).

Men reported beginning masturbation at an earlier age than women (*Z* = 7.50, *P* < .001, Cohen’s *d* = 0.40), engaging in a higher maximum number of masturbations per day (*Z* = 8.91, *P* < 0.001, Cohen’s *d* = 0.44), as well as masturbating more frequently in the last month (*χ^2^*(4) = 93.16, *P* < .001, *ϕ_c_* = 0.30).

With respect to dyadic sexual activity, men rated their first sexual intercourse higher than women (*Z* = 6.30, *P* < .001, Cohen’s *d* = 0.36). Furthermore, men reported a greater number of sexual partners since sexual initiation, within the past year, and in the last 30 days, and a higher number of dyadic sexual intercourse in the last 30 days.

Next, we examined gender differences in questionnaires scores. Table 3 shows means and standard deviations for women and men separately, along with the results of between-gender comparisons (Mann Whitney *U* test). The analyses revealed that women scored higher than men on the BES (*Z* = 5.51, *P* < .001, Cohen’s *d* = 0.30) and the anxiety subscale of the Hospital Anxiety and Depression Scale (HADS; *Z* = 7.46, *P* < .001, Cohen’s *d* = 0.39). Conversely, men scored significantly higher than women on the CSBD-19 (*Z* = 9.62, *P* < .001, Cohen’s *d* = 0.52) and the BPS (*Z* = 10.66, *P* < .001, Cohen’s *d* = 0.57) than women.

**Table 3:** Results obtained in questionnaires divided by gender.

	**Women** **(n = 787)**	**Men** **(n = 740)**	
	***M* (SD)**		**Mann–Whitney *U* (Cohen’s *d*)**
**Binge Eating Screener**	8.19 (8.29)	5.92 (6.71)	**5.51** ^*^ **(0.30)**
**Compulsive Sexual Behavior Disorder-19**	**n = 745** 30.62 (9.31)	**n = 706** 35.83 (10.60)	**9.62** ^*^ **(0.52)**
**Brief Pornography Screen**	**n = 599** 1.32 (2.20)	**n = 661** 2.76 ( 2.78)	**10.66** ^*^ **(0.57)**
**Hospital Anxiety and Depression Scale** Anxiety Depression	8.11 (4.27) 5.45 (3.78)	6.49 (4.00) 5.30 (3.80)	**7.46** ^*^ **(0.39)** 0.87 (0.04)

^*^ *P* < .001

Additionally, positive correlations were observed between the total BES score and all other questionnaires, both for the entire sample and for women and men separately. For the overall sample, the strongest correlation was with the HADS depression subscale (*r*_total sample_ = 0.47, *P* < .001), followed by the HADS anxiety subscale (*r*_total sample_ = 0.46, *P* < .001), BPS (*r*_total sample_ = 0.25, *P* < .001), and CSBD-19 (*r*_total sample_ = 0.21, *P* < .001). The results of the bivariate correlations between all analyzed questionnaires are presented in Table 4.

**Table 4:** Pearson correlations between total scores obtained in Binge Eating Screen (BES), Brief Pornography Screen (BPS), and Compulsive Sexual Behavior Disorder-19 (CSBD-19), and results obtained in Hospital Anxiety and Depression Scale (HADS) subscales.

	**BES total**	**BPS total**	**CSBD-19 total**	**HADS anxiety**	**HADS depression**
**BES total score** **All** **Women** **Men**		0.25^*^ 0.26^*^ 0.39^*^	0.21^*^ 0.18^*^ 0.35^*^	0.46^*^ 0.44^*^ 0.46^*^	0.47^*^ 0.48^*^ 0.46^*^
**BPS total score All Women Men**			0.53^*^ 0.35^*^ 0.59^*^	0.23^*^ 0.22^*^ 0.37^*^	0.23^*^ 0.16^*^ 0.32^*^
**CSBD-19 total score All Women Men**				0.18^*^ 0.13^*^ 0.35^*^	0.24^*^ 0.17^*^ 0.33^*^
**HADS anxietyAll Women Men**					0.71^*^ 0.72^*^ 0.72^*^
**HADS depression All Women Men**					

^*^ *P* < .001.

To assess the prevalence and co-occurrence of BED and CSBD symptoms, we divided the sample based on cutoff scores of the questionnaires dedicated to measuring the severity of given symptoms. For the BES, a cutoff value of 17 was used to classify participants into low-risk (BED−) and high-risk (BED+) groups, with 186 participants exceeding this threshold. Similarly, for the CSBD-19 scale, a cutoff score of 50 was employed to categorize participants into low-risk (CSBD−) and high-risk (CSBD+) groups, with 84 participants exceeding this threshold.

A cross-tabulation analysis was then conducted to compare the frequency of BED and CSBD across the distinct groups (see Tables 5 and 6). The analysis revealed significant differences between BED and CSBD co-occurrence in the overall sample (*χ^2^*(1) = 35.97, *P* < .001, *ϕ_c_* = 0.16), and among men (*χ^2^*(1) = 59.00, *P* < .001, *ϕ_c_* = 0.29). However, no significant difference was observed in women (*χ^2^*(1) = 1.51, *P* = .22, *ϕ_c_* = 0.05).

**Table 5:** Prevalence of CSBD symptoms among participants with BED.

	**CSBD−**	**CSBD+**
	**n (%)**	
**BED−** All Women Men	1224 (95.6%) 621 (97.5%) 603 (93.6%)	57 (4.4%) 16 (2.5%) 41 (6.4%)
**BED+** All Women Men	143 (84.1%) 103 (95.4%) 40 (64.5%)	27 (15.9%) 5 (4.6%) 22 (35.5%)
**All (N = 1451)** **Women (n = 745)** **Men (n = 706)**	1367 (94.2%) 724 (97.2%) 643 (91.1%)	84 (5.8%) 21 (2.8%) 63 (8.9%)

**Table 6:** Prevalence of BED symptoms among participants with CSBD.

	**BED−**	**BED+**
	**n (%)**	
**CSBD−** All Women Men	1224 (89.5%) 621 (85.8%) 603 (93.8%)	143 (10.5%) 103 (14.2%) 40 (6.2%)
**CSBD+** All Women Men	57 (67.9%) 16 (76.2%) 41 (65.1%)	27 (32.1%) 5 (23.8%) 22 (34.9%)
**All (N = 1451)** **Women (n = 745)** **Men (n = 706)**	1281 (88.3%) 637 (85.5%) 644 (91.2%)	170 (11.7%) 108 (14.5%) 62 (8.8%)

Finally, we compared CSBD symptoms between participants in the BED− and BED+ groups (see Table 7). The analysis showed that a significantly higher percentage of participants in the BED+ group (as compared to the BED− group) reported difficulty controlling their sexual behavior (*χ^2^*(1) = 55.60, *P* < .001, *ϕ_c_* = 0.19) and sought help for loss of control over sexual behavior in the past (*χ^2^*(1) = 43.21, *P* < .001, *ϕ_c_* = 0.17).

**Table 7:** Various aspects of sexual behavior and related factors divided by scores obtained in BES.

	**BED−** **(n = 1341)**	**BED+** **(n = 186)**	**Mann–Whitney *U* (Cohen’s *d*)**	***χ*** ^***2***^ **(Cramér’s *V*)**
**Have you ever had difficulty controlling your sexual behavior? (n, %)** Yes No	181, 13.5% 1160, 86.5%	65, 34.9% 121, 65.1%	–	55.60^***^ (0.19)
**Types of out-of-control behavior (yes/no) (n, %)** Pornography consumption Excessive masturbation Paid sexual services Casual sexual activity Excessive fantasizing Erotic chats and cameras Other	**n = 181** 97, 53.6% 90, 49.7% 8, 4.4% 30, 16.6% 92, 50.8% 22, 12.2% 20, 11.0%	**n = 65** 26, 40.0% 30, 46.2% 6, 9.2% 10, 15.4% 31, 47.7% 5, 7.7% –	–	3.53 (0.12) 0.24 (0.03) 2.06 (0.09) 0.05 (0.01) 0.19 (0.03) 0.98 (0.06) –
**Have you ever sought any form of help due to a loss of control over sexual behavior? (n, %)** Yes No	14, 1% 1327, 99%	15, 8.1% 171, 91.9%	–	43.21^***^ (0.17)
**Type of help used due to sexual behavior (n, %)** Psychological Sexological Self-help groups Religious Psychiatric Other	**n = 14** 6, 42.9% 9, 64.3% 3, 21.4% 6, 42.9% 1, 7.1% 1, 7.1%	**n = 15** 5, 45.5% 5, 33.3% 2, 6.9% 1, 6.7% 3, 20.0% 1, 6.7%	–	0.28 (0.10) 2.78 (0.31) 0.33 (0.11) 5.18^*^ (0.42) 1.01 (0.19) 0.003 (0.01)
**Taking medication for sexual difficulties (n, %)**	**n = 670** 57, 8.5%	**n = 70** 10, 14.3%	–	2.57 (0.06)
**Onset of first pornography exposure (M; SD)** Has never watched pornography	**n = 1109** 17.27; 7.35 **n *=* 232**	**n = 151** 16.62; 6.99 **n *=* 35**	1.134 (0.09)	–
**Sexual experiences with partner before pornography (n, %)** Cuddling/Foreplay Masturbation Vaginal contact (intravaginal) Anal contact (intrarectal) Oral–genital contact (French love) Has had no experience	**n = 1109** 261, 23.5% 205, 18.5% 145, 13.1% 22, 2% 92, 8.3% 656, 59.2%	**n = 151** 36, 23.8% 46, 30.5% 17, 11.3% 3, 2.0% 13, 8.6% 77, 51%	–	0.01 (0.002) 11.95^***^ (0.10) 0.39 (0.02) 0.00 (0.00) 0.02 (0.004) 3.64 (0.05)
**Frequency of pornography use in the last year (n, %)** Not at all Once a month or less 2-4 times a month 2-3 times a week 4 times a week or more	**n = 1109** 475, 42.8% 282, 25.4% 160, 14.4% 107, 9.6% 85, 7.7%	**n = 151** 56, 37.1% 38, 25.2% 21, 13.9% 21, 13.9% 15, 9.9%	–	4.31 (0.06)
**Time spent on pornography during the last 7 days (in minutes) (M; SD)** Has not watched pornography last week	**n = 552** 36.96; 64.64 **n *=* 557**	**n = 87** 40.64; 63.48 **n *= 64***	0.55 (0.06)	–
**On average, how many times have you viewed pornography in the last month? (M; SD)**	**n = 1109** 3.75; 7.77	**n = 151** 4.95; 9.59	1.67 (0.14)	–
**Average duration of a single pornography session in the last month (in minutes) (M; SD)**	**n = 596** 17.70; 16.71	**n = 90** 20.10; 30.33	0.23 (0.10)	–
**Age of first masturbation (M; SD)** Has never masturbated	**n = 913** 15.65; 4.99 **n *=* 428**	**n = 129** 15.35; 5.78 **n *=* 57**	0.90 (0.06)	–
**Maximum number of masturbations per day (M; SD)**	**n = 913** 2.71; 2.25	**n = 129** 3.56; 3.41	3.32^***^ (0.29)	–
**Frequency of masturbation in the last month (n, %)** Not at all Once a month or less 2 to 4 times a month 2 to 3 times a week 4 times a week or more	**n = 913** 308, 33.7% 200, 21.9% 213, 23.3% 121, 13.3% 71, 7.8%	**n = 129** 28, 21.7% 30,23.3% 35, 27.1% 19, 14.7% 17, 13.2%	–	9.94^*^ (0.10)
**Onset of first sexual intercourse (M; SD)** Never had sexual intercourse	**n = 1178** 19.39; 3.70 **n *=* 163**	**n = 153** 19.30; 4.74 **n *= 33***	0.94 (0.02)	–
**Subjective rate of the first sexual intercourse (from 1 – unsuccessful to 10 – very successful) (M; SD)**	**n = 1178** 5.74; 2.75	**n = 153** 5.14; 2.78	2.44^*^ (0.22)	–
**Number of sexual partners since sexual initiation (M; SD)**	**n = 1178** 6.58; 11.51	**n = 153** 7.84; 13.34	0.60 (0.10)	–
**Number of sexual partners during the last year (M; SD)**	**n = 1178** 0.91; 2.47	**n = 153** 1.18; 2.02	1.58 (0.12)	–
**Number of dyadic sexual intercourse in the last 30 days (M; SD)**	**n = 1178** 2.63; 5.19	**n = 153** 2.03; 2.78	0.87 (0.14)	–
**Number of sexual partners in the last 30 days (M; SD)**	**n = 1178** 0.80; 1.57	**n = 153** 0.80; 1.47	1.32 (0.00)	–

^***^ *P* < .001; ^**^P < .01; ^*^P < .05

Additionally, a significant difference was observed between the groups regarding the type of help sought (*χ^2^*(1) = 5.18, *P* < .05, *ϕ_c_* = 0.42), with more participants in the BED− group utilizing religious forms of support compared to the BED+ group. In the case of sexual experiences with a partner before exposure to pornography, significantly more participants in the BED− group reported masturbation compared to those in the BED+ group (*χ^2^*(1) = 11.95*, P* < .001, *ϕ_c_* = 0.10).

Furthermore, the BED+ group reported a higher maximum number of masturbation per day (*Z* = 3.32, *P* < .001, Cohen’s *d* = 0.29), and a greater frequency of masturbation in the past month (*χ^2^*(4) = 9.94, *P* < .05, *ϕ_c_* = 0.10) compared to the BED− group. Lastly, participants in the BED− group rated their first sexual intercourse higher than those in the BED+ group (*Z* = 2.44, *P* < .05, Cohen’s *d* = 0.22).

## Discussion

In the current study, we have addressed significant gap in the literature by examining the prevalence and co-occurrence of BED and CSBD. Along with their associations with sexual behavior and levels of anxiety and depression, in a representative Polish sample.

The data revealed that men reported higher CSBD symptom scores compared to women and were more likely to experience difficulties controlling sexual behavior, including excessive pornography use, masturbation, and engagement with erotic chats or webcams, consistent with prior findings that CSBD is more prevalent in men.^21^ Notably, there was a substantial discrepancy between self-perceived loss of control over sexual behavior and meeting the diagnostic threshold for CSBD, particularly among women (11.4% vs 2.8%) compared to men (21.1% vs 8.9%).

Gender differences also emerged in developmental patterns, with men initiating masturbation and pornography use at earlier ages and reporting more sexual experiences prior to first pornography exposure, whereas women were more likely to have had no prior sexual experience. These differences may reflect distinct developmental pathways shaping subsequent sexual behaviors across genders.

Men and women in our study exhibited distinct profiles, aligning with literature showing higher CSBD symptoms among men^8^^,^^19^ and higher anxiety and BED symptoms among women.^16^ While BED and CSBD symptoms frequently co-occurred in men, this pattern was absent in women, indicating potential gender-specific mechanisms. Women may express compulsive sexual behaviors interpersonally rather than through solitary activities typically assessed in CSBD measures, leading to underreporting due to stigma and measurement limitations.^21^^,^^22^ Additionally, emotional dysregulation and stress vulnerability may underlie BED in women, while trauma and mood disorders may be more closely linked to CSBD.^22^ In contrast, men’s higher impulsivity and reward sensitivity^23^^,^^24^ may contribute to co-occurring BED and CSBD through shared mesolimbic dopamine pathway hyperactivity, while women may use binge eating primarily for emotional coping.^25^

Help-seeking patterns further differed by gender, with men more likely to seek assistance for compulsive sexual behaviors, whereas stigma may inhibit women from reporting or seeking help.^22^ Existing CSBD assessment tools, which emphasize solitary sexual behaviors, may inadequately capture women’s experiences, leading to underestimation of CSBD prevalence and its co-occurrence with BED. These findings highlight the need for gender-sensitive, culturally informed assessment approaches to capture the complexity of BED and CSBD across genders.^22^

Positive correlations between BED symptoms and measures of anxiety, depression, and CSBD underscore the interplay between BED and psychological distress. Given the established associations of both BED and CSBD with other psychiatric disorders,^7^^,^^10^^,^^11^ comprehensive assessment and treatment approaches should address co-occurring conditions.

Our findings demonstrate a significant co-occurrence of BED and CSBD among men, with BED+ individuals reporting greater difficulties controlling sexual behavior and higher help-seeking rates, suggesting shared underlying mechanisms such as impulsivity and heightened reward sensitivity that may exacerbate sexual behavior dysregulation.

From a neurophysiological perspective, both BED and CSBD have been linked to dysregulation in the brain’s reward system, particularly hyperactivity in the mesolimbic dopamine pathway involved in reward anticipation and reinforcement.^26-28^ Heightened neural responses to rewarding stimuli, such as food or sexual content, may contribute to compulsive consumption patterns observed in BED+ and CSBD+ individuals. Gender differences in neural responses may further clarify behavioral patterns, with men exhibiting greater sensitivity to visual sexual stimuli driven by ventral striatum activity, while women’s higher anxiety and BED symptoms may be linked to insula and anterior cingulate cortex activity, regions associated with emotional processing and interoception.

The observed shared mechanisms underlying BED and CSBD, such as heightened impulsivity, reward sensitivity, and emotional dysregulation, have important clinical implications. They suggest that transdiagnostic treatment approaches may be effective for individuals experiencing both conditions. Interventions such as dialectical behavior therapy (DBT) and cognitive behavioral therapy (CBT), which target emotion regulation, impulse control, and maladaptive coping strategies, have shown efficacy in treating BED^29^^,^^30^ and may be adapted for CSBD symptoms as well. Additionally, mindfulness-based interventions and acceptance and commitment therapy (ACT) may help individuals increase awareness of urges and reduce compulsive behaviors across both eating and sexual domains.^31^ Addressing co-occurring anxiety and depression within these interventions is also crucial, as these factors may exacerbate compulsive behaviors.

Overall, these findings emphasize the complex interplay between gender, neurophysiology, and sociocultural factors in shaping the prevalence, expression, and co-occurrence of BED and CSBD, underscoring the need for development and application of integrated, transdiagnostic treatment models that address both conditions within a unified therapeutic framework.

### Limitations

The results of this study should be interpreted considering several limitations. First, its cross-sectional design precludes drawing conclusions about causality. To better understand the relationship between BED and CSBD and their development over time, longitudinal studies are needed. Second, the study relied on self-reported data, which may be subject to biases such as social desirability or recall errors. Additionally, the use of nonvalidated measures for some sexual behavior variables may have introduced further inconsistencies and inaccuracies. Future research would benefit from incorporating validated instruments and integrating multiple data sources, including behavioral or physiological measures, to help mitigate these biases and improve the reliability and validity of findings. Third, the questions assessing sexual behavior were not derived from a validated scale, which may affect the consistency and comparability of these measurements. Finally, while this study provides insights into the prevalence and co-occurrence of BED and CSBD within a representative Polish sample, it did not specifically focus on the influence of cultural factors such as societal attitudes toward sex and food, or the potential impact of religious beliefs and traditions on the manifestation and reporting of compulsive behaviors. These cultural elements are recognized as important contextual factors that may shape both the experience of symptoms and patterns of help-seeking but were not systematically assessed in the current research. It is worth noting, however, that cultural context may play a particularly relevant role in the self-identification and reporting of CSBD or PPU symptoms. For example, a large-scale international study,^32^ conducted across 42 countries, demonstrated substantial cross-national variability in PPU prevalence, with rates ranging from 3.2% to 16.6% depending on the country. In that study, the prevalence of PPU in Poland was estimated at 9.09%, highlighting the potential influence of sociocultural norms and values. Future studies are encouraged to incorporate culturally sensitive measures and investigate how cultural and religious contexts influence the development, presentation, and management of BED and CSBD, thereby providing a more comprehensive understanding of these disorders in Poland and beyond.

## Conclusions

This study highlights significant gender-based differences in sexual behaviors, psychological profiles, and the co-occurrence of CSBD and BED. Beyond behavioral observations, the findings underscore the need to explore the psychopathological and neurophysiological mechanisms underpinning these conditions, offering insights into shared vulnerabilities and their clinical implications. The present results support the notion that BED and CSBD, while distinct diagnoses, may reflect overlapping mechanisms—such as reward dysregulation and impulse control deficits—particularly in men. As hypothesized, BED and CSBD symptoms frequently co-occurred and were positively associated with anxiety and depression, reinforcing the role of affective dysregulation in both conditions. Furthermore, consistent with our expectations, men were more likely to report CSBD symptoms and show co-occurrence with BED, while women exhibited higher BED severity and anxiety symptoms. These gendered patterns highlight the importance of considering sex-specific psychological, biological, and sociocultural dynamics when assessing and treating these disorders.
